# Bronchial artery-pulmonary artery fistula with pulmonary embolism and massive hemoptysis: a rare case report and literature review

**DOI:** 10.3389/fmed.2025.1678443

**Published:** 2025-12-01

**Authors:** Guiyu Lu, Juncai Wu, Cuidong Deng

**Affiliations:** Department of General Practice, Zigong Fourth People’s Hospital, Zigong, China

**Keywords:** anticoagulants, pulmonary embolism, embolization therapeutic, vascular fistula, case report

## Abstract

**Background:**

Bronchial artery-pulmonary artery fistula is a rare vascular anomaly, either congenital or acquired due to recurrent infections. When associated with pulmonary embolism and massive hemoptysis, it significantly increases the risk of mortality and poses complex therapeutic challenges. Only two such cases have been reported in the literature based on PubMed and CNKI database. Anticoagulation therapy requires careful management to balance thrombotic and hemorrhagic risks.

**Case presentation:**

A 60-year-old male who presented with recurrent massive hemoptysis was diagnosed with a bronchial artery–pulmonary artery fistula and pulmonary embolism, confirmed by computed tomography pulmonary angiography and digital subtraction angiography. Hemostasis was achieved through bronchial artery embolization, followed by individualized anticoagulation therapy with warfarin, adjusted according to INR monitoring.

**Conclusion:**

This case underscores the importance of a personalized diagnostic and therapeutic approach in managing the coexistence of vascular fistula, pulmonary embolism, and hemoptysis. Bronchial artery embolization remains the cornerstone of acute bleeding control, while a carefully tailored and closely monitored anticoagulation strategy is essential to reduce thrombotic risk without increasing the likelihood of hemorrhagic complications.

## Introduction

Massive hemoptysis, defined as 300–600 mL of bleeding within 24 h or any life-threatening amount causing airway obstruction, accounts for approximately 5% of hemoptysis cases and carries a mortality rate of 6.5%–38% (1). Common causes include bronchiectasis, tuberculosis, aspergillosis, necrotizing pneumonia, and lung cancer, with about 20% of cases being idiopathic (1). Bronchial artery to pulmonary artery fistula (BPF), a rare vascular malformation, may arise from congenital dysplasia, trauma, or chronic infections (2). The abnormal high-pressure shunt from bronchial to pulmonary arteries often leads to cough and dyspnea, and accounts for 2.6%–13% of massive hemoptysis cases based on retrospective studies (3). Due to its rarity and non-specific symptoms, BPF is frequently misdiagnosed. Pulmonary embolism (PE), a common cardiopulmonary emergency, is associated with a 30-day all-cause mortality rate of 5.4%, with 1.7% directly attributable to PE (4).

The concurrence of BPF, PE, and massive hemoptysis is exceedingly rare, with only two cases reported in PubMed and CNKI databases as of July 2025. This triad significantly increases mortality risk and complicates management, as anticoagulation for PE may exacerbate hemoptysis. Hypothesized mechanisms, such as abnormal blood flow in BPF predisposing to thrombus formation, may contribute to this rare presentation. In the absence of standardized guidelines, tailored diagnostic and therapeutic strategies based on imaging and risk assessment are required. This report presents a case of BPF with concurrent PE and massive hemoptysis, accompanied by a literature review of associated diagnostic and therapeutic challenges.

## Case report

### Proband’s clinical details

In August 2023, a 60-year-old male presented to the emergency department with a sudden recurrence of hemoptysis, producing approximately 7–10 mL of fresh blood per episode during physical exertion one hour prior to arrival. The patient had a known 4-year history of bronchiectasis, previously managed with intermittent anti-infective and hemostatic therapies, which had effectively controlled symptoms. He was a non-smoker and reported no family history of malignancy or chronic diseases, and no psychosocial issues affecting his condition or treatment adherence. On admission, the patient complained of throat irritation, shortness of breath, and palpitations, which he described as “an overwhelming tightness in the chest.” Vital signs were as follows: body temperature 37.2 °C, respiratory rate 20 breaths/min, heart rate 94 beats/min, blood pressure 148/92 mmHg, and peripheral oxygen saturation of 95% on room air. Physical examination revealed coarse breath sounds over the left lung field and diminished breath sounds accompanied by faint rales in the right lung field. The patient expressed anxiety regarding the recurrence, noting that this episode felt “more intense” and “less controllable” than previous ones, which prompted his immediate visit to the hospital.

### Initial examination and treatment

**FIGURE 1:**
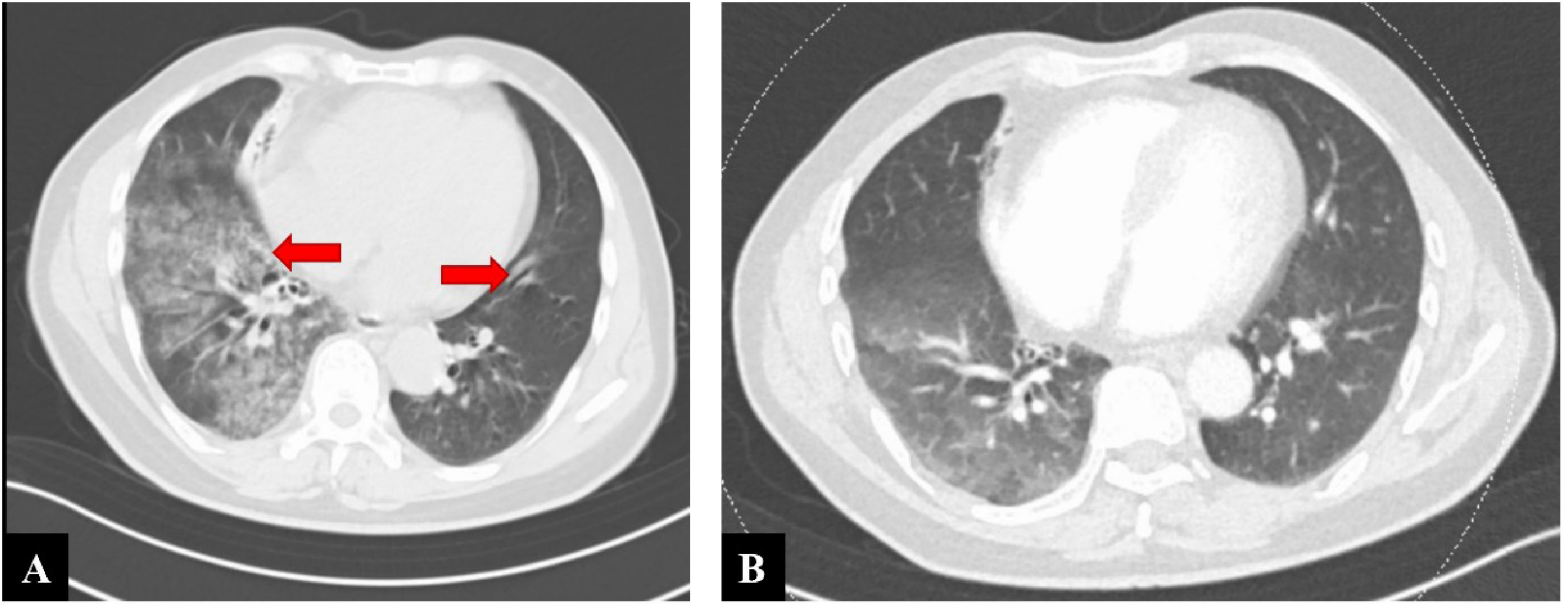
**(A)** Chest computed tomography (CT) on admission during massive hemoptysis, showing multiple ground-glass opacities in the lower lobes of both lungs (red arrows). **(B)** Follow-up chest CT after treatment, showing significant resolution of the opacities with no new hemorrhagic lesions.

Laboratory tests revealed a white blood cell count of 4.81 × 10∧9/L, hemoglobin of 123 g/L, platelet count of 93 × 10∧9/L, and C-reactive protein of 0.4 mg/L. Emergency chest computed tomography (CT) showed multiple patchy ground-glass opacities in both lower lobes, suggestive of hemorrhage or inflammation, along with bronchiectasis and atelectasis in the right middle and lower lobes (Figure 1A). Initial treatment included oxygen therapy, intravenous hemostatic agents (arginine vasopressin, hemocoagulase, carbazochrome, and aminomethylbenzoic acid), and levofloxacin for suspected infection. Lower-limb ultrasonography revealed acute distal deep vein thrombosis (DVT) confined to the right gastrocnemius and bilateral calf muscular veins, with hypoechoic intraluminal thrombi, absent Doppler flow, mild venous dilatation, and no proximal extension, indicating an elevated risk of PE. Due to severe hemoptysis, anticoagulation was deferred, and hemocoagulase and aminomethylbenzoic acid were discontinued to minimize thrombotic risk while prioritizing bleeding control. Platelet counts normalized from hospital day 6 and remained stable (120–210 × 10∧9/L).

### Further examination and treatment

**FIGURE 2:**
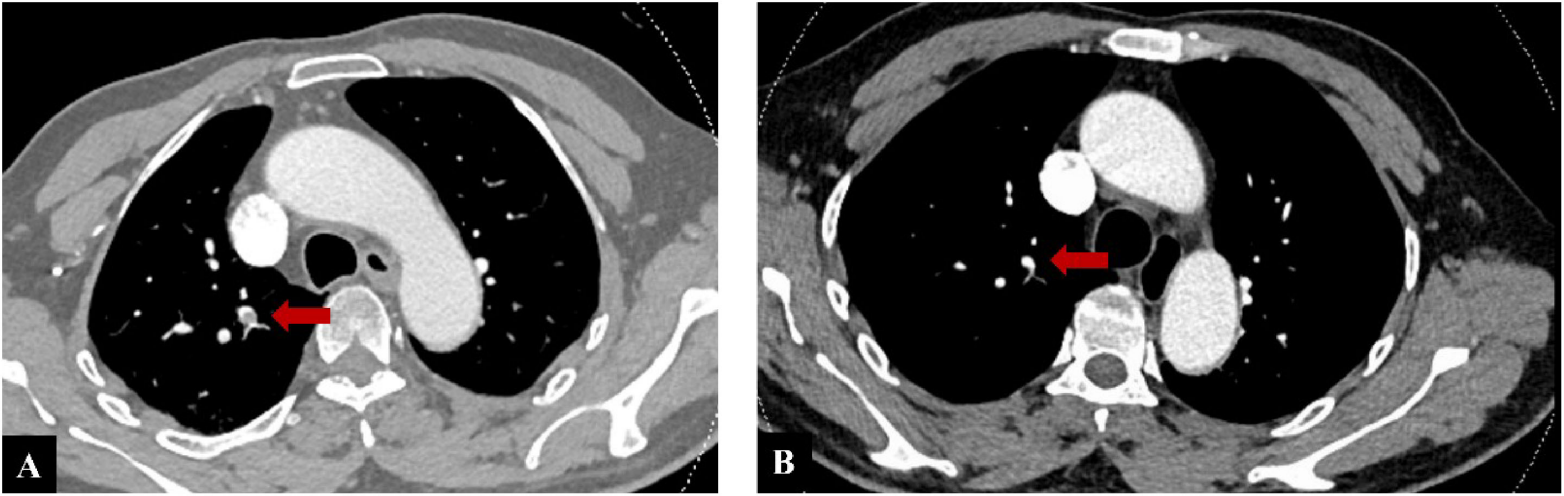
**(A)** Computed tomography pulmonary angiography (CTPA) on admission, red arrow demonstrating thrombus formation in the right pulmonary artery. **(B)** Follow-up CTPA at the 6-month evaluation, demonstrating complete disappearance of the right pulmonary artery thrombus.

**FIGURE 3:**
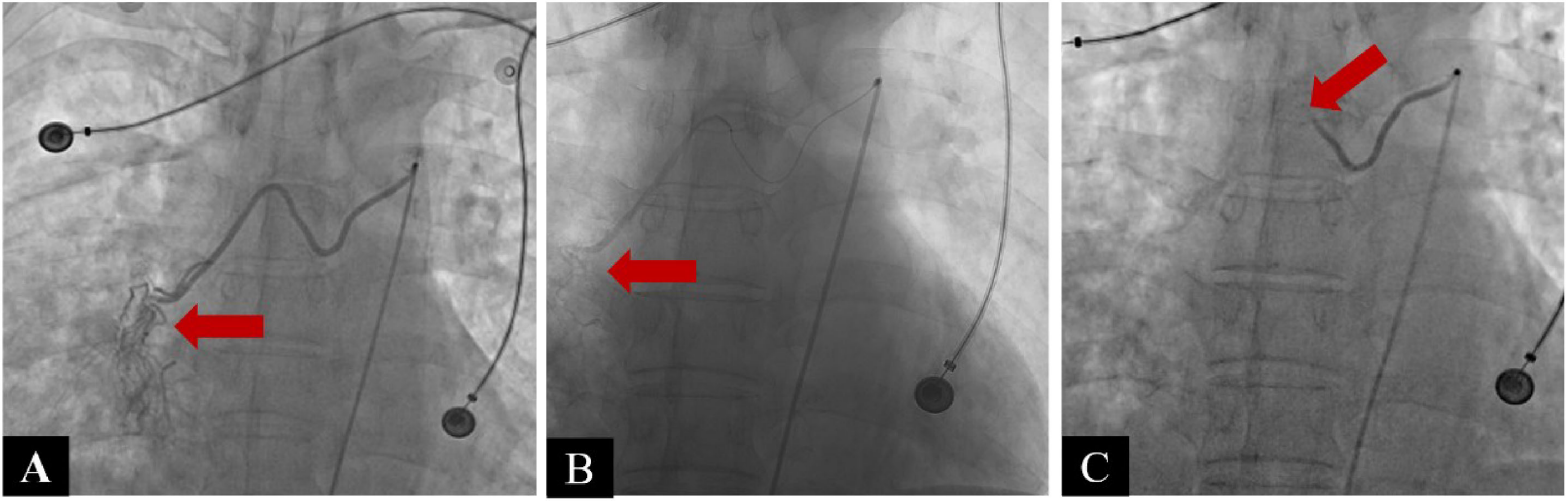
Bronchial artery angiography and embolization for bronchial artery to pulmonary artery fistula (BPF). **(A)** Pre-embolization digital subtraction angiography (DSA) of the right bronchial artery shows tortuous bronchial branches with systemic-to-pulmonary shunting, consistent with BPF (red arrow). **(B)** Super-selective distal catheterization of the right bronchial artery using a micro-catheter, demonstrating staining of the distal arterial segment. **(C)** Post-embolization DSA demonstrates occlusion of the abnormal vessels with resolution of BPF.

The patient’s hemoptysis escalated to 400–500 mL daily. Emergency CT pulmonary angiography (CTPA) revealed filling defects in the pulmonary arteries of the right middle and lower lobes, confirming PE (Figure 2A). Following urgent interventional consultation, digital subtraction angiography (DSA) was performed under general anesthesia. DSA revealed hypertrophy, tortuosity, and angulation of the broncho-intercostal trunk, common bronchial arteries, and right phrenic artery, with increased, disorganized vascular proliferation and a BPF in the right lung (Figure 3A). Super-selective bronchial artery embolization achieved immediate hemostasis.

### Anticoagulation decision

Warfarin was initiated on day 10 after embolization once hemostatic stability had been met (≥72 h without hemoptysis, stable hemodynamics and hemoglobin/hematocrit, no new hemorrhagic lesions on follow-up CT, and no active extravasation or residual high-flow shunting on post-embolization angiography; Figures 1B, 3C). Considering the patient’s elevated risk of bleeding, heparin bridging was deliberately avoided. Warfarin was started at an initial dose of 1.25 mg/day and titrated to maintain an international normalized ratio (INR) of 2.0–3.0, target near 2.5 to minimize bleeding risk. The patient demonstrated good adherence to regular INR follow-ups.

### Outcome and follow-up

During the 2-year follow-up (until July 2025), no recurrence of hemoptysis or thrombosis was observed. At the 6-month evaluation, duplex ultrasonography confirmed complete resolution of the lower-extremity thrombus, and CTPA demonstrated complete disappearance of the right pulmonary artery thrombus (Figure 2B). Anticoagulation was discontinued thereafter. Subsequent duplex ultrasonography remained negative for DVT, although no further CTPA was performed. The patient reported relief after symptom resolution and remained positive about his recovery and long-term outcome.

## Discussion

Treatment for this patient was challenging due to the coexistence of two life-threatening conditions—BPF and PE—with persistent massive hemoptysis. Initial imaging, including CTA, identified only PE, while subsequent DSA confirmed BPF. This rare combination is prone to misdiagnosis or missed diagnosis. While anticoagulation is essential for reducing sudden death risk in PE, it is contraindicated in massive hemoptysis, complicating treatment decisions significantly. Risk stratification showed an sPESI score of 0 (low PE-related mortality), but active massive hemoptysis conferred high bleeding risk despite a HAS-BLED of 1. In assessing bleeding risk, a platelet count of ∼93 × 10∧9/L is seldom associated with spontaneous major bleeding, and most invasive procedures target ≥40–50 × 10∧9/L (5); counts normalized from hospital day 6, supporting hemostatic stability. These factors justified delaying anticoagulation, prioritizing BAE to control bleeding, and omitting heparin bridging when warfarin was initiated after stabilization.

Bronchial artery–pulmonary artery fistula is rare and may be congenital or acquired secondary to tuberculosis, HIV, trauma, or malignancy (2). In our patient, congenital BPF was unlikely. Contrast-enhanced CTA and catheter angiography showed hypertrophied bronchial arteries within a bronchiectatic lobe forming a fistulous connection to subsegmental pulmonary arterial branches. No features of congenital anomalies (e.g., proximal interruption of the pulmonary artery) were identified. This pattern in diseased parenchyma is typical of acquired systemic-to-pulmonary arterial shunts (6). Screening for hereditary hemorrhagic telangiectasia using the Curaçao criteria was negative, and no pulmonary arteriovenous malformations were detected; per international guidelines, germline testing is reserved for clinically suspected cases (7). Congenital BPFs are classically described in younger patients or lungs without concomitant inflammatory disease, further supporting an acquired, bronchiectasis-related BPF here (8). Repeated infections can remodel bronchial arteries with tortuosity and aneurysmal change, predisposing to abnormal systemic–pulmonary shunts that may rupture and cause massive, and potentially fatal hemoptysis (9). BPF is a recognized but often overlooked cause of occult massive hemoptysis (10).

Early diagnosis of BPF is challenging because chest X-rays and CT scans often non-specific; as the disease progresses, atelectasis or pneumonia may develop (11). Although CTA can reveal bronchial artery abnormalities, false negatives have been reported (11), and misdiagnosis as isolated PE can delay therapy and increase mortality (12). DSA remains the gold standard, allowing both diagnosis and simultaneous embolization. In unexplained massive hemoptysis, DSA should be prioritized to localize the source and guide timely intervention. Given the rarity of concomitant BPF and PE and the absence of specific guidelines, we prioritized embolization to secure hemostasis before starting anticoagulation.

Introduced clinically in 1973, Bronchial artery embolization (BAE) is now the first-line treatment for massive hemoptysis (13). It achieves immediate hemostasis in 77%–100% of cases, though recurrence remains high (9%–44%), usually at 1–2 or 12–24 months (13, 14), often related to comorbidities, vascular fistulas, or inappropriate embolic materials (13). To reduce ischemia and nerve injury, particles larger than 200–250 μm for distal embolization are recommended. Commonly, PVA particles (300–500 μm) perform well, and additional embolic agents such as gelatin sponge, thrombin, and glue are frequently utilized (13, 15). Combining materials (e.g., coils with PVA) further improves success rates (15). In this case, considering that the bronchial artery may share a common origin with the anterior spinal artery and communicate with intercostal and mediastinal branches, overly small particles could penetrate too distally and reflux into non-target vessels, thereby increasing the risk of spinal ischemia. To address this, 350–560 μm PVA particles were selected for durable distal occlusion, while 560–710 μm gelatin sponge particles were added for proximal flow control. This achieved rapid, temporary occlusion, reducing collateral recruitment and preserving the option of re-intervention. Their absorbable nature also conferred cost-effectiveness. Because selective bronchial artery catheterization was technically challenging, protective embolization of the intercostal arteries with 3 mm and 2 mm coils was undertaken to reduce non-target embolization risk. Final angiography showed distal occlusion of abnormal vessels and resolution of the BPF (Figure 3B). This layered, combined embolic strategy balance safety and efficacy. Nevertheless, unrecognized collaterals may still lead to severe complications, such as bronchial wall necrosis or spinal ischemia (13), highlighting the importance of meticulous imaging and careful monitoring, especially in patients with complex vascular anatomy.

Deep vein thrombosis in the lower limbs is considered the most common cause of PE. Registry studies report a 3-month mortality rate up to 17% after venous thrombosis diagnosis (16). The coexistence of BPF and PE is extremely rare, with only two prior cases reported. One Japanese case involved a patient with chronic right pulmonary artery thrombosis and a pulmonary vascular anomaly presenting with massive hemoptysis, who improved following surgery (17). Another Chinese report described a patient with recurrent hemoptysis initially diagnosed with PE. Anticoagulation worsened the hemoptysis, and further angiography revealed a BPF. Bleeding was controlled through BAE, though the report did not specify a follow-up anticoagulation strategy (18) (Table 1). With no American College of Chest Physicians/European Society of Cardiology guidance specific for BPF with PE, management should be individualized. Most reports prioritize controlling life-threatening hemoptysis with interventional methods and initiating anticoagulation only after stabilization (19, 20). Other strategies include surgery, bronchoscopic hemostasis, and the use of coils or NBCA glue have been described (21), while temporary inferior vena cava filters or thrombectomy may serve as bridging options when anticoagulation must be delayed (22). However, no clear recommendations exist on the selection or timing of anticoagulants, as early administration risks severe bleeding, while delays increase the risk of thrombus progression or recurrence.

**TABLE 1:** Comparison of clinical features, management, and outcomes among three bronchial artery to pulmonary artery fistula (BPF) cases complicated by pulmonary thromboembolism/hemoptysis.

Case	Our case	Japanese case (17)	Chinese case (18)
Demographics	60-year-old male	62-year-old female	65-year-old male
Presentation	Recurrent massive hemoptysis	Recurrent hemoptysis	Recurrent massive hemoptysis
Diagnosis	BPF, PE, and DVT	Chronic thromboembolic pulmonary hypertension, BPF	Bilateral bronchial artery malformation, BPF, PE
Key imaging	CTPA: acute PE (right middle/lower lobes). DSA: hypertrophied, tortuous bronchial arteries with systemic-to-pulmonary shunt/fistula. Lower-limb ultrasonography: acute distal DVT	Contrast-enhanced CT: thrombus in right pulmonary artery; hypertrophied bronchial artery draining into right lower-lobe pulmonary artery (BPF)	DSA: markedly enlarged bronchial artery at T4 (≈3×), tortuous distal course, common trunk to multiple lobes, contrast extravasation; right-sided BPF, pulmonary embolism (right pulmonary artery trunk and branches)
Hemodynamics	PE risk stratification low (sPESI 0); post-hemostasis clinical stability; 6-months CTPA: complete thrombus resolution	Right-heart catheterization: PAP 61/21 (mPAP 34) mmHg; PVR 408 dyn⋅s⋅cm^−5^; post-op hemodynamics improved	PAP rose rapidly ∼48→*83*→108 mmHg; decreased after embolization
Hemostasis strategy	Layered BAE: distal PVA 350–560 μm (durable occlusion) + proximal gelatin sponge 560–710 μm (flow control); protective intercostal coiling as needed	Surgery: right thoracotomy pulmonary endarterectomy + ligation of bronchial artery (single-stage)	BAE: micro-coils + gelatin sponge
Anticoagulation	Warfarin initiated day 10 after hemostasis; no heparin bridging; INR target 2.0–3.0 (≈2.5); stopped at 6 months (imaging-confirmed resolution)	Not reported	LMWH → aggravated bleeding → stopped; rivaroxaban (post-hemostasis) → recurrent hemoptysis → stopped; BAE → definitive hemostasis; long-term regimen not specified
Outcomes	Immediate hemostasis; thrombus (DVT/PE) disappeared at 6 months; no recurrence over 2 years	Hemoptysis resolved; hemodynamics improved after pulmonary endarterectomy	Hemoptysis resolved post-embolization; PAP decreased; no further details on long-term outcome

Direct oral anticoagulants (DOACs) reduce venous thrombosis recurrence and show efficacy comparable to warfarin for PE without routine monitoring, which favors their use in low- to moderate-risk PE (23). However, DOACs have a higher bleeding risk (24), limited reversal access, and higher cost in resource-limited settings (25); evidence in high-bleeding-risk patients remains insufficient (23). LMWH acts rapidly but is only partially reversible with protamine, limiting its use in severe bleeding (26). In contrast, warfarin’s anticoagulant effect can be rapidly reversed with vitamin K, fresh frozen plasma, or prothrombin complex concentrate (24), which are widely accessible and offer a safety advantage in complex bleeding cases. After BAE, we initiated warfarin 10 days post-procedure. Reports suggest anticoagulation can often be resumed within days after BAE (mean 4.9 ± 3.5 days in a CTEPH cohort) (27) and as early as the next day once hemostasis is secure (19); broader guidance supports restarting within 1–2 weeks after major bleeding (28). In our patient, 6 months of warfarin achieved complete thrombus resolution, and no hemoptysis recurred over a 2-year follow-up.

This case may provide practical insights for managing the rare concurrence of BPF, PE, and massive hemoptysis. We prioritized hemostasis with BAE, ensured short-term stability, and then initiated a reversible oral anticoagulant without heparin bridging. This approach while resolved thrombosis avoiding rebleeding. The case illustrates a potential framework—control bleeding, confirm stability, then anticoagulate—that could guide care in settings where evidence is limited. Our layered embolization strategy supported durable hemostasis and made timed anticoagulation feasible. Although limited to a single case, these observations highlight the lack of unified recommendations and the need for shared management guidelines through future multicenter studies. Given inter-patient variability, the findings should be interpreted with caution.

## Conclusion

This report describes a rare case of BPF complicated by PE and massive hemoptysis. The favorable outcome following staged hemostasis and cautious anticoagulation underscores the need for individualized management, while highlighting the absence of standardized guidelines for such rare conditions.

## Data Availability

The original contributions presented in this study are included in this article/supplementary material, further inquiries can be directed to the corresponding author.
